# Evolution of surgical management and functional outcomes of craniopharyngiomas: a systematic review and meta-analysis

**DOI:** 10.1007/s10143-026-04308-8

**Published:** 2026-04-30

**Authors:** Federico Valeri, Simone Antonio de Sanctis, Gianluca Trevisi, Jacopo Ciccani, Sabrina Chiloiro, Antonella Giampietro, Antonio Bianchi, Rosalinda Calandrelli, Ciro Mazzarella, Simona Gaudino, Mario Rigante, Marco Gessi, Pier Paolo Mattogno, Liverana Lauretti, Francesco Doglietto

**Affiliations:** 1 Department of Neurosurgery, Fondazione Policlinico Universitario Agostino Gemelli IRCCS, Rome, Italy; 2 Facoltà di Medicina e Chirurgia, Università Cattolica del Sacro Cuore, Rome, Italy; 3 Department of Endocrinology and Metabolism, Pituitary Unit, Fondazione Policlinico Universitario Agostino Gemelli IRCCS, Rome, Italy; 4 Department of Neurosciences, Imaging and Clinical Sciences, G. D’Annunzio University Chieti-Pescara, Chieti, Italy; 5 Neurosurgical Unit, Ospedale Spirito Santo, Pescara, Italy; 6 Radiology and Neuroradiology Unit, Department of Imaging, Radiation Therapy and Hematology, Fondazione Policlinico Universitario Agostino Gemelli IRCCS, Rome, Italy; 7 Department of Otolaryngology and Head-Neck Surgery, Fondazione Policlinico Universitario Agostino Gemelli IRCCS, Rome, Italy; 8 Department of Woman and Child Health Sciences and Public Health, Anatomic Pathology Unit, Fondazione Policlinico Universitario Agostino Gemelli IRCCS, Roma, Italy; 9 Department of Neurosurgery, Mater Olbia Hospital, Olbia, Italy

**Keywords:** Craniopharyngioma, Endoscopic endonasal, Hypothalamic syndrome, Obesity, Pituitary, Transcranial

## Abstract

Craniopharyngiomas (CPs) are rare benign epithelial tumors, whose management remains one of the most challenging feats in skull base surgery. Over recent decades, the evolution from transcranial microsurgical approaches (TCA) to extended endoscopic endonasal approaches (EEEA) has reshaped their management. This systematic review and meta-analysis aimed to elucidate the evolution of surgical treatment and define the functional outcomes of adult CP patients. A systematic review of the literature was carried out according to PRISMA 2020 guidelines, including papers from PubMed, Scopus, and Ovid databases. Meta-analyses of proportions were conducted using random-effects models (REML estimator), with heterogeneity assessed via the I² statistic. Meta-regression analyses explored the influence of publication year and geographic origin. Thirty-one retrospective studies encompassing 1,855 adult patients met the inclusion criteria. The mean age clustered between the fourth and fifth decades, with balanced sex distribution and follow-up durations ranging from 12 to 126 months. Preoperatively, anterior hypopituitarism occurred in 61.5%, diabetes insipidus (DI) in 19.7%, and obesity in 21.3% of patients. The majority of tumors were suprasellar-intraventricular (88.9%), with adamantinomatous histology predominating (72.2%). EEEA was the most used technique (57.4%), followed by TCA (35.6%). Gross total resection (GTR) rate was 68.1%, increasing to 76.2% in the EEEA subgroup. Meta-regression confirmed a significant temporal trend toward increased EEEA utilization (*p* = 0.040), while GTR rates remained stable over time. Postoperatively, hypopituitarism affected 79.6%, permanent DI 54.7%, and obesity 42.2% of patients; cognitive deficits were present in 10.6%. Recurrence occurred in 14.4% and all-cause mortality was 1.9%. The country of origin significantly influenced GTR outcomes (*p* = 0.004), reflecting institutional and technical variability. Egger’s test indicated no publication bias for EEEA rates (*p* = 0.683) but confirmed asymmetry for GTR (*p* < 0.001). The EEEA route is being increasingly favored for midline and suprasellar CPs, achieving robust rates of GTR (76.2%) for midline and suprasellar CPs, with higher visual improvement rates and acceptable morbidity in experienced hands. However, rates of postoperative endocrine and hypothalamic dysfunction remain high, highlighting the need for hypothalamic-sparing strategies and individualized surgical planning. Underreporting of hypothalamic invasion limits accurate interpretation of functional outcomes. Future prospective multicentric studies integrating molecular data, standardized neuropsychological assessment and clinical and surgical data reporting are essential to refine surgical decision-making and optimize quality of life for CP patients.

## Introduction

Craniopharyngiomas (CPs) are rare, histologically benign epithelial tumors believed to originate from remnants of Rathke’s pouch [1], accounting for approximately 1–3% of all primary intracranial neoplasms [2]. Despite their benign nature, they present a formidable surgical challenge due to their intimate relationship with the optic apparatus, pituitary stalk, hypothalamus, and major cerebral vessels [3]. The dual goal of achieving durable tumor control while preserving functional outcomes continues to define their management as one of the most debated areas in contemporary neurosurgery. Traditionally, transcranial microsurgical approaches (TCA) were considered the mainstay of treatment [4–6], allowing direct visualization of the suprasellar region and third ventricle. However, these approaches were associated with significant postoperative morbidity, such as hypothalamic injury, hypopituitarism, and metabolic alterations [7], and visibility may be hindered when CPs develop under the optic chiasm or upward inside the ventricle [8]. Over the past two decades, the evolution of endoscopic endonasal techniques has revolutionized CP’s surgical landscape. The extended endoscopic endonasal approach (EEEA) offers a direct midline trajectory to the suprasellar and third ventricular compartments without brain retraction, enabling improved visualization of tumor–hypothalamic interfaces and bimanual dissection under panoramic view [9]. Many studies have since reported encouraging outcomes with EEEA, showing gross-total resection (GTR) rates comparable to TCAs, but with superior visual recovery and lower neurovascular manipulation [10]. Nonetheless, the risk of postoperative endocrinological dysfunction remains substantial [11], and the balance between radical tumor removal and functional preservation continues to drive surgical decision-making. Moreover, technical heterogeneity, differences in tumor topography, and institutional expertise have contributed to the wide variability in reported outcomes across the literature. Furthermore, emerging molecular insights, such as the identification of BRAF V600E mutations in papillary CPs, as well as the efficacy and safety of modern radiation techniques [12], have opened new therapeutic options that complement management algorithms [13]. In this context, the present systematic review aims to provide an updated synthesis of the surgical evolution, radicality, and functional outcomes of adult CP management. Integrating data from the most recent and rigorous retrospective studies, this research’s objective is to elucidate temporal trends, identify determinants of surgical success, and outline the persistent challenges and future directions in the neurosurgical treatment of these complex lesions.

## Methods

### Systematic review

**Fig. 1:**
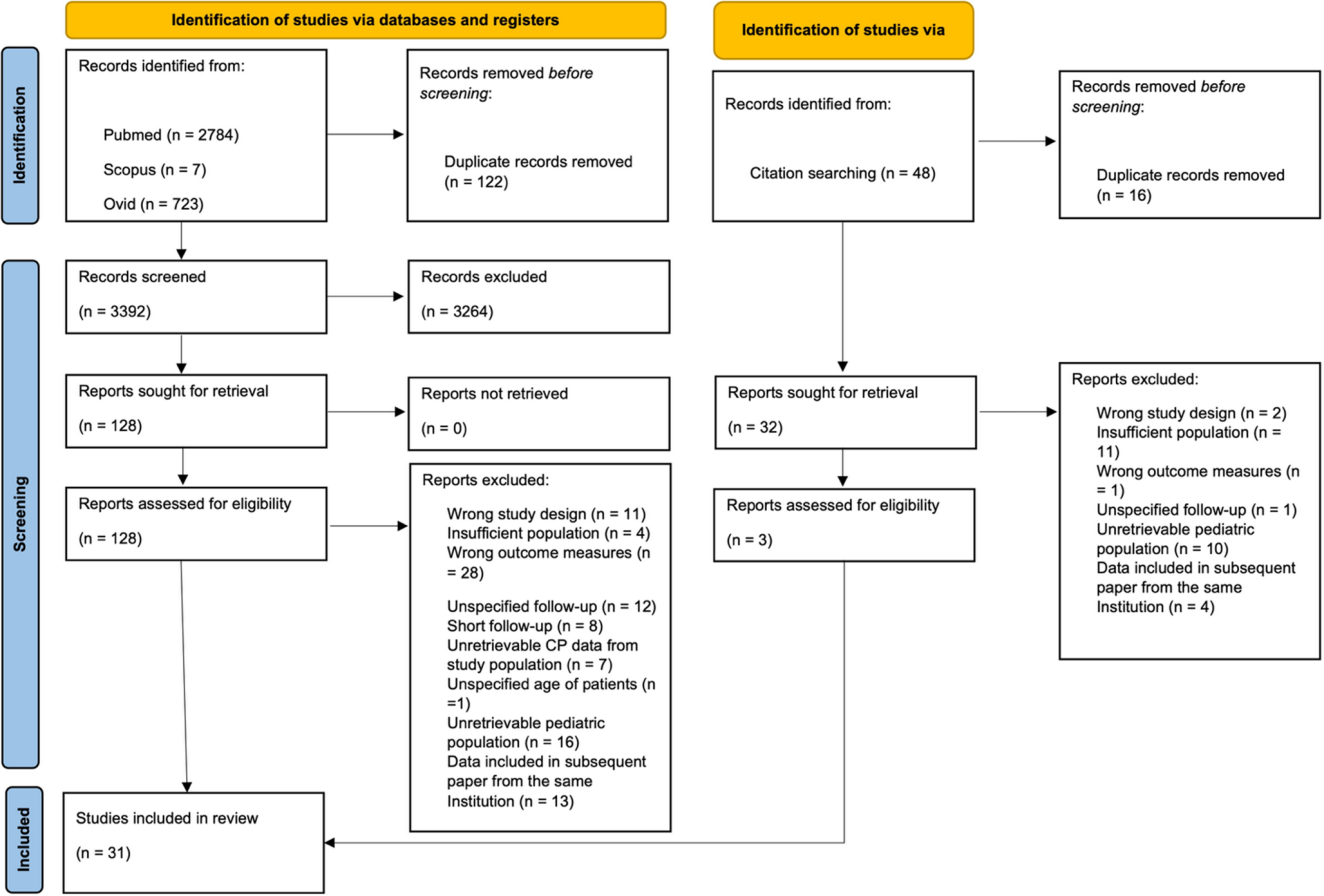
PRISMA diagram of the systematic review

This review was carried out according to the updated Preferred Reporting Items for Systematic Reviews and Meta-Analysis (PRISMA 2020) guidelines [14] (Fig. 1). A computer-aided search of Pubmed, Scopus, and Ovid databases was performed to identify relevant studies. A search string was developed for each database to extract papers focusing on neuroendocrinological outcomes after CP surgery. A combination of the following search terms was used: “craniopharyngioma”, “hypothalamic”, “pituitary”, “obesity”, “behavior”, “behavioral”, “deficit”, “dysfunction”, “syndrome”, “change”, “hypopituitarism”, “diabetes insipidus”. The following terms were used to exclude studies irrelevant to the current review: “pediatric”, “children”, and “childhood”.

All papers published until October 21 st 2025 were considered. Titles and abstracts were screened independently by two reviewers (FV and SDS) against a set of pre-defined eligibility criteria. We included original studies in English reporting at least 10 patients operated for CPs. To be included, each study had to report: (1) post-puberal population (age > 15 years); (2) a minimum follow-up of 12 months from surgery; (3) at least one of the primary outcome measures. Case reports, reviews, meta-analyses, letters to the editor, conference abstracts, book chapters, laboratory or animal investigations, and papers based on epidemiological registries were excluded.

Eligible studies were screened for full-text analysis, and disagreements were resolved by consensus or appeal to a third senior reviewer (SC). If two or more papers belonged to the same Institution, only the most recent and/or complete was included.

An Excel sheet was developed to extract data on the following variables: study details, sample size, demographics, preoperative and postoperative neuroendocrinological findings (hypopituitarism, diabetes insipidus, obesity, and hypothalamic cognitive deficit), tumor topography, histological diagnosis, surgical specifics (approach, infiltration of hypothalamus by the tumor, pituitary stalk sacrificing), surgical outcomes (extent of resection, recurrences/progressions, disease-related deaths) and follow-up time.

Primary outcome measures considered were:

1. postoperative anterior hypopituitarism (defined as at least one deficient hormonal axis);
2. diabates insipidus (DI);
3. overweight (defined as BMI≥25 kg/m^2^);
4. hypothalamic cognitive impairment (comprising behavioral changes, depression, memory loss, daytime drowsiness, and attention disorders).

Secondary variables studied were recurrence and disease-related death rates. All variables were considered at the last available follow-up.

### Statistical analysis

All meta-analytic procedures were performed using JASP software (JASP Team, University of Amsterdam, Netherlands) and OpenMeta[Analyst] software (Brown University, Providence, RI, USA). Given the anticipated variability in study designs and populations, a random-effects model was employed for all analyses to provide a conservative estimate of the pooled effects. The Restricted Maximum Likelihood (REML) estimator was utilized to calculate the between-study variance. For single-arm meta-analyses of proportions including the prevalence of symptoms, resection rates, and tumor characteristics, raw data were transformed using the logit transformation to approximate a normal distribution for pooling, and subsequently back-transformed for reporting as percentages with 95% confidence intervals. Comparative analyses between surgical approaches were conducted using the Odds Ratio as the summary statistic. Heterogeneity among included studies was assessed using the I^2^ statistic and Cochran’s Q test, with I2 values greater than 50% indicating substantial heterogeneity. To investigate potential sources of heterogeneity, meta-regression analyses were performed using the year of publication as a continuous moderator and the country of origin as a categorical moderator. Comparisons between preoperative and postoperative morbidity rates were conducted using a mixed-effects meta-regression framework, with the timepoint treated as a categorical moderator to assess statistical significance. A p-value of less than 0.05 was considered statistically significant for all tests.

### Publication bias

Publication bias was evaluated visually using funnel plots and statistically quantified using Egger’s regression test. A qualitative assessment of the publication bias was conducted with the ROBINS-I V2 tool [15].

## Results

### Study selection and demographic characteristics

The systematic literature search yielded a final inclusion of 31 retrospective observational studies [16–46] published predominantly between 2018 and 2025. The selected studies represented a global distribution of neurosurgical centers, with significant contributions from institutions in China, Italy, the United States, France, Germany, Australia, Poland, South Korea, India, Russia, and Japan. The cumulative sample size comprised 1855 post-puberal patients treated for craniopharyngioma, with study-specific mean ages consistently ranging from the early 30 s to the 70 s, though the aggregate mean age across cohorts clustered between the fourth and fifth decades of life. Gender distribution varied by center but remained relatively balanced in the aggregate analysis. The mean duration of postoperative follow-up varied across the included studies, ranging from 12 months to over 126 months, providing a substantial longitudinal window for assessing outcomes (Table 1).

### Preoperative clinical presentation

Quantitative synthesis of baseline clinical characteristics revealed a high prevalence of endocrinological and metabolic dysfunction before surgical intervention. Preoperative pituitary hormone deficiency was the most frequently reported comorbidity, with a pooled prevalence of 61.5% (95% CI: 53.0–70.0%; I^2^ = 88.72%). Central diabetes insipidus was present at diagnosis in a smaller but clinically significant subset of patients, affecting 19.7% (95% CI: 13.9–25.5%; I^2^ = 89.79%) of the pooled cohort. Regarding metabolic status, preoperative overweight status was identified in 21.3% (95% CI: 15.4–27.2%; I^2^ = 96.82%) of the population. Furthermore, evaluation of hypothalamic functional integrity revealed that 13.2% (95% CI: 8.0–18.3%; I^2^ = 72.25%) of patients presented with detectable cognitive deficits before surgery, underscoring the neurocognitive impact of the lesion on surrounding structures (Table 1).

**Table 1:** Anagraphic and preoperative data extracted from studies (Preop = preoperative)

First author, year	Anagraphic data				Preoperative clinical data				Tumor Localisation	
	Patients (*n*)	Age (years)	Sex (Male/Female)	Follow-up (months)	Preop anterior hypopituitarism (*n*)	Preop DI (*n*)	Preop overweight (*n*)	Preop cognitive impairment (*n*)	Sellar/suprasellar localisation (*n*)	Suprasellar/intraventricular localisation (*n*)
Bao Y.Y., 2025[16]	193	48	111/82	59,5	140	19	0	13	15	178
Bove I., 2025[17]	61	51,9	34/27	79,13	31	7	24	6	0	61
Guo Y., 2025[18]	390	43	215/175	12	247	99	NA	NA	100	290
Ke D., 2025[19]	16	42,9	NA	31	15	3	NA	NA	NA	NA
Gaillard S., 2024[20]	79	41,5	33/46	62,1	65	29	0	23	33	46
Chen Y., 2023[21]	31	44	19/12	39	21	9	0	7	0	31
Gallotti A. L., 2022[22]	59	43,5	33/26	66,4	NA	14	13	NA	NA	NA
Godil S. S., 2022[23]	39	56,1	NA	68,4	20	10	NA	NA	NA	NA
Zhou Y., 2022[24]	14	46,4	8/6	26,2	5	1	3	3	0	14
Zamora R. E., 2022[25]	68	43,9	42/26	38,6	NA	15	44	8	1	67
Zoli M., 2022[26]	50	54,3	25/25	43	29	15	NA	NA	0	50
Chen Z., 2021[27]	73	39	38/35	19	58	25	1	4	0	73
Duan D., 2021[28]	45	53	23/22	26	NA	8	34	NA	NA	NA
Iglesias P. N., 2021[29]	53	72,3	26/27	46,7	NA	2	13	NA	2	51
Giese H., 2020[30]	71	48,9	39/32	69,3	23	1	25	14	19	52
Simonin A., 2020[31]	16	42,9	9/7	22,1	4	6	NA	1	0	16
Cai M., 2019[32]	22	47	10/12	54,8	17	5	NA	NA	0	22
Mou J., 2019[33]	54	42	NA	22	26	7	5	NA	13	41
Dho Y. S., 2018[34]	68	50	34/34	30,7	51	1	NA	21	0	68
Zielinski G., 2018[35]	30	40,5	11/19	73,8	20	7	4	NA	0	30
Bal E., 2016[36]	12	31,7	7/5	60,1	8	2	NA	NA	1	11
Fomichev D., 2016[37]	136	49,3	60/76	42	NA	NA	NA	28	0	136
Kshettry V. R., 2016[38]	43	42,3	25/18	40,4	25	6	3	4	NA	NA
Lee E. J., 2015[39]	82	42	53/29	46	20	10	NA	10	NA	NA
Han S., 2014[40]	29	35	9/20	76,5	23	10	NA	0	0	29
Kunihiro N., 2014[41]	16	47,9	6/10	63,6	10	9	NA	5	0	16
Wang L., 2014[42]	16	32,2	7/9	50	10	2	NA	NA	16	0
Jane J. A. Jr., 2010[43]	12	50,77	5/7	13,3	10	3	NA	NA	5	7
Jung T. Y., 2009[44]	39	45,8	25/14	126,7	12	5	NA	2	NA	NA
Gardner P. A., 2008[45]	16	55	10/6	34	12	3	NA	NA	0	16
Nishizawa S., 2006[46]	22	48,9	15/7	70,8	18	0	NA	1	0	22
Total (n)	1855	Median 45,8	932/814	Median 46	920	333	169	150	205	1327

### Tumor topography and histopathology

Meta-analytic pooling of tumor topography data revealed a marked predominance of lesions involving the floor of the third ventricle. Tumors classified as suprasellar-intraventricular constituted the vast majority of the studied cases, with a pooled estimate of 88.9% (95% CI: 84.7–93.2%; I^2^ = 97.02%). Conversely, lesions strictly categorized as sellar-suprasellar were less frequently represented in this specific cohort selection, accounting for 11.1% (95% CI: 6.8–15.3%; I^2^ = 97.02%) (Table 1). Histopathological analysis confirmed the distribution of subtypes anticipated in an adult population. The adamantinomatous subtype was the dominant pathology, observed in 72.2% (95% CI: 67.3–77.1%; I^2^ = 76.53%) of cases, while the papillary subtype comprised 23.9% (95% CI: 18.7–29.2%; I^2^ = 83.07%) of the histologically verified tumors (Table 2).

**Table 2:** Surgical and histological data extracted from studies (3VF = third ventricle floor; ACP = adamantinomatous; microTNS = microscopic transsphenoidal approach; PCP = papillary)

First author, year	Surgical Approach					Intraoperative findings		Extent of Resection			Histology			
	EEA (*n*)	TCA (*n*)	MicroTNS (*n*)	Ventriculoscopy (*n*)	Other (*n*)	3VF infiltration (*n*)	Stalk sacrifice (*n*)	GTR (*n*)	NTR (*n*)	STR (*n*)	ACP (*n*)	PCP (*n*)	Mixed (*n*)	Undetermined (*n*)
Bao Y.Y., 2025[16]	193	0	0	0	0	NA	NA	173	13	7	166	27	0	0
Bove I., 2025[17]	61	0	0	0	0	NA	NA	40	8	13	53	8	0	0
Guo Y., 2025[18]	101	289	0	0	0	NA	NA	318	0	72	272	118	0	0
Ke D., 2025[19]	16	0	0	0	0	NA	NA	16	0	0	NA	NA	NA	NA
Gaillard S., 2024[20]	66	13	0	0	0	46	15	26	44	9	62	17	0	0
Chen Y., 2023[21]	12	19	0	0	0	NA	14	26	2	3	11	20	0	0
Gallotti A. L., 2022[22]	39	0	0	0	0			31	0	8	31	8	0	0
Godil S. S., 2022[23]	14	0	0	0	0	NA	0	13	1	0	5	9	0	0
Zhou Y., 2022[24]	0	41	17	5	5	NA	NA	NA	NA	NA	49	4	0	15
Zamora R. E., 2022[25]	36	10	0	4	0	NA	NA	NA	NA	NA	NA	NA	NA	NA
Zoli M., 2022[26]	73	0	0	0	0	NA	20	51	16	6	NA	NA	NA	NA
Chen Z., 2021[27]	NA	NA	NA	NA	NA	NA	NA	10	0	35	33	7	0	5
Duan D., 2021[28]	0	39	20	0	0	NA	NA	51	2	6	49	10	0	0
Iglesias P. N., 2021[29]	9	51	0	3	4	NA	NA	30	0	41	54	8	0	9
Giese H., 2020[30]	22	28	3	0	0	NA	NA	0	24	29	28	23	2	0
Simonin A., 2020[31]	16	0	0	0	0	NA	NA	10	0	6	NA	NA	NA	NA
Cai M., 2019[32]	0	22	0	0	0	3	6	19	0	3	14	8	0	0
Mou J., 2019[33]	54	0	0	0	0	NA	NA	41	3	10	NA	NA	NA	NA
Dho Y. S., 2018[34]	0	30	0	0	0	NA	9	30	0	0	NA	NA	NA	NA
Zielinski G., 2018[35]	68	0	0	0	0	NA	35	62	0	6	NA	NA	NA	NA
Bal E., 2016[36]	12	0	0	0	0	NA	NA	10	0	2	9	3	0	0
Fomichev D., 2016[37]	136	0	0	0	0	NA	NA	98	0	38	109	27	0	0
Kshettry V. R., 2016[38]	43	0	0	0	0	38	22	19	13	11	29	14	0	0
Lee E. J., 2015[39]	2	79	0	0	1	NA	49	71	7	3	62	19	0	1
Han S., 2014[40]	0	29	0	0	0	16	NA	23	0	6	23	6	0	0
Kunihiro N., 2014[41]	0	16	0	0	0	NA	10	9	6	1	NA	NA	NA	NA
Wang L., 2014[42]	0	0	16	0	0	0	0	3	13	0	9	2	0	5
Jane J. A. Jr., 2010[43]	12	0	0	0	0	NA	NA	9	1	2	7	4	0	1
Jung T. Y., 2009[44]	NA	NA	NA	NA	NA	NA	15	24	0	15	29	10	0	0
Gardner P. A., 2008[45]	16	0	0	0	0	NA	NA	7	3	6	NA	NA	NA	NA
Nishizawa S., 2006[46]	0	22	0	0	0	NA	20	20	0	2	NA	NA	NA	NA
Total (n)	1001	688	56	12	10	103	215	1240	156	340	1104	352	2	36

## Surgical management and resection rates

Regarding the surgical strategy, the quantitative analysis focused exclusively on the EEEA and TCA, as data regarding microscopic transsphenoidal surgery and primary ventriculostomy were anecdotal within the included cohorts. The EEEA was the predominant technique utilized, with a pooled prevalence of 57.4% (95% CI: 42.9–72%; I^2^= 99.88%). TCAs were employed in a slightly smaller proportion of the aggregate population, accounting for 35.6% (95% CI: 27.5–45.5%; I^2^ = 99.7%) of the surgical procedures. A direct comparison of the two approaches yielded a pooled odds ratio of 6.824 (95% CI: 1.588–29.312), indicating a statistically significant preference and likelihood for the endoscopic route.

**Fig. 2:**
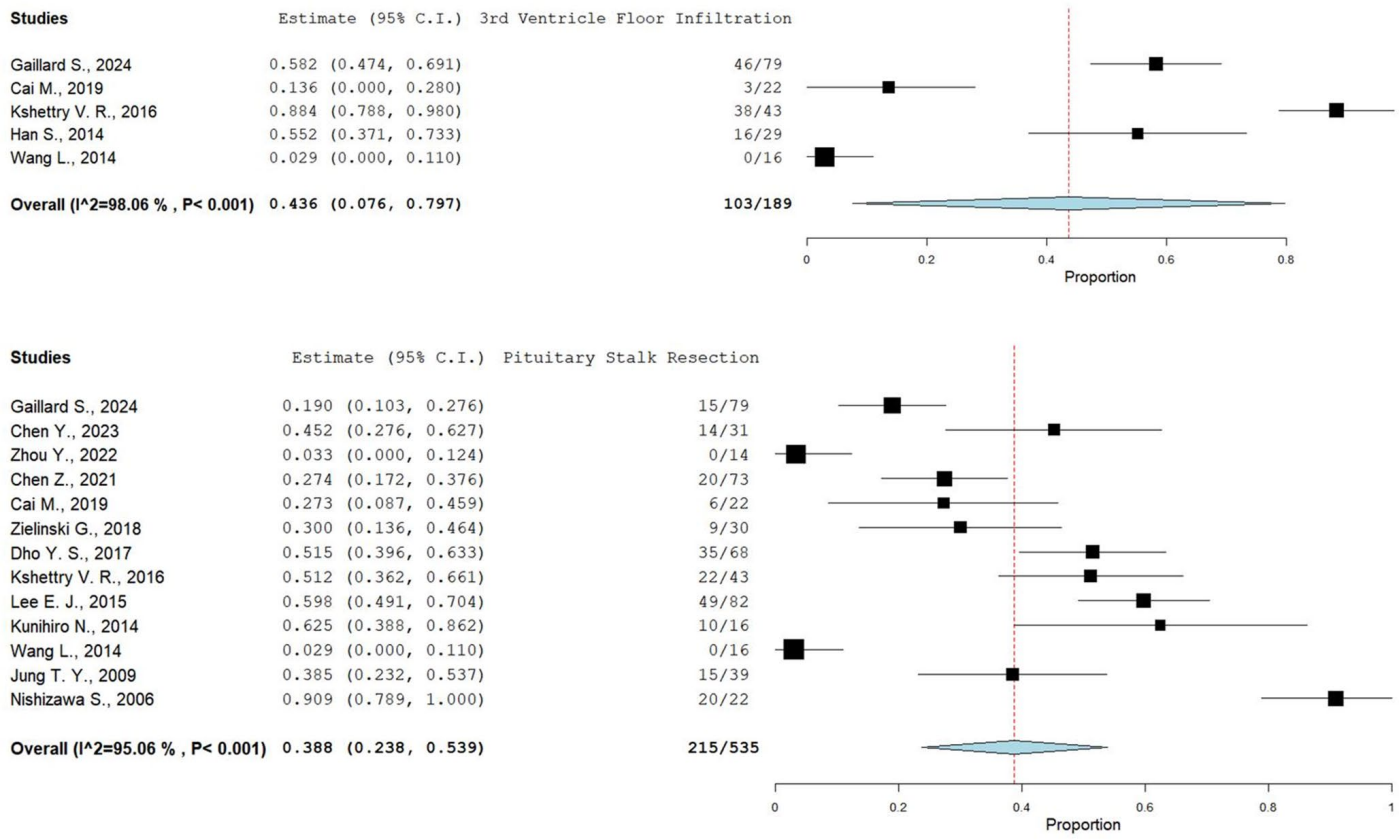
Forest-Plot diagram of included studies reporting third ventricle floor invasion (upper) and pituitary stalk resection (lower). A marked heterogeneity is visible, reflecting the underreporting of the data and the different populations (e.g., different localizations) included in the studies

Intraoperative details were poorly reported. Indeed, only 5 Authors [20, 32, 38, 40, 42] reported data about infiltration of the third ventricle floor, which was infiltrated in 43.6% (95%CI: 7.6–79.7%). Other 13 Authors [20, 21, 24, 27, 32, 34, 35, 38, 39, 41, 42, 44, 46] (reported that pituitary stalk resection was performed in 38.8% (23.8%−53.9%) of cases, with high heterogeneity among studies (I^2^ = 95%). (Fig. 2)

In terms of surgical efficacy, the pooled rate of GTR across all approaches was 68.1% (95% CI: 52.4–83.7%). Subgroup analysis of the EEEA specifically demonstrated a robust GTR rate of 76.2% (95% CI: 68.6–83.8%) (Table 2).

### Meta-regression analysis

**Fig. 3:**
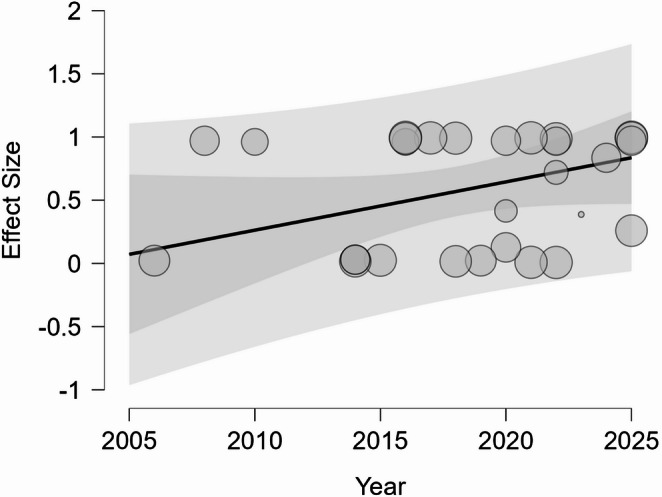
Meta-regression bubble plot demonstrating the temporal trend of the EEA. The X-axis represents the year of publication, and the Y-axis represents the effect size (logit-transformed proportion). The size of each bubble is proportional to the study weight. The regression line (positive slope) indicates a statistically significant increase in the utilization of EEA over the study period (*p* = 0.040). A logit value of 0 corresponds to a 50% utilization rate; values greater than 0 indicate a predominance of the endoscopic approach

Given the substantial heterogeneity observed in the chosen surgical approach and extent of resection, a meta-regression analysis was conducted to identify potential sources of variability, utilizing the year of publication and the country of origin as moderators. For the EEEA, the analysis revealed a statistically significant positive association between the year of publication and the utilization of the technique (Q_M_=4.229, *p* = 0.040, coefficient 0.152), confirming a temporal trend toward increased adoption of the endoscopic route in recent years (Fig. 3). Conversely, the rate of GTR did not exhibit a significant correlation with the year of publication (*p* = 0.614), suggesting that resection efficacy has remained relatively stable over the studied period. However, the country of origin was identified as a significant moderator for GTR (Q_M_=27.659, *p* = 0.004). This finding suggests that geographical location—likely serving as a proxy for center volume, surgical philosophy, or regional expertise—significantly contributes to the variance in reported resection outcomes, whereas it does not significantly influence the choice of surgical approach.

### Postoperative outcomes and follow-up

The assessment of postoperative morbidity revealed a significant burden of endocrinological and hypothalamic dysfunction following surgical intervention.

Pituitary function was heavily impacted, with a pooled prevalence of postoperative hypopituitarism reaching 79.6% (95% CI: 74.8–84.4%). Statistical comparison confirmed a significant deterioration compared to the preoperative status (*p*<0.001), indicating that the vast majority of patients required long-term hormone replacement therapy.

Disturbance of water balance was also frequent, with permanent DI affecting 54.7% (95% CI: 45.8–63.5%) of the cohort, a statistically significant increase from baseline (*p*<0.001).

**Fig. 4:**
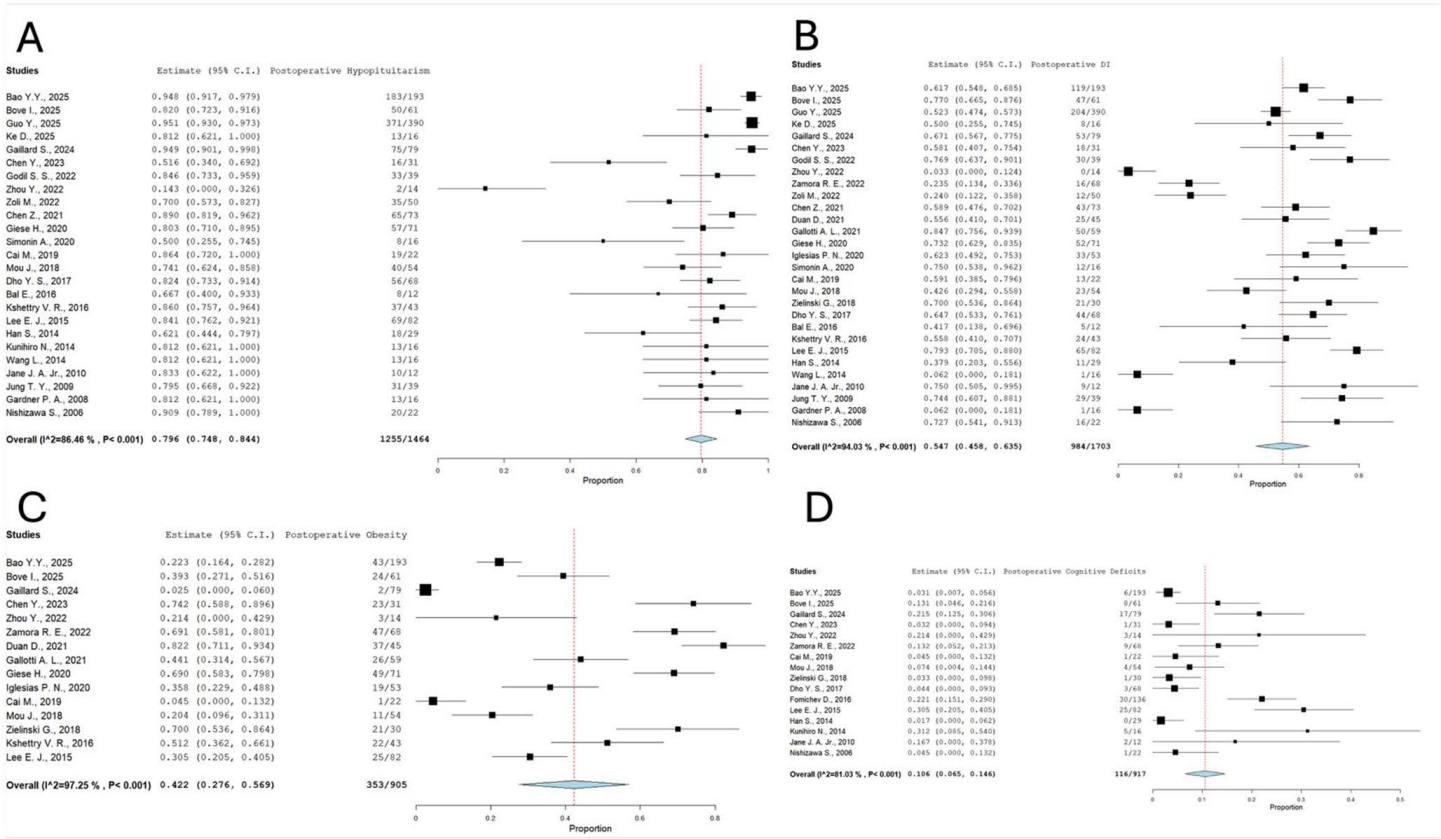
Forest-plot diagram of postoperative rates of hypopituitarism (**A**), DI (**B**), overweight (**C**) and cognitive impairment (**D**). Various degrees of heterogeneity are visible in reported outcomes, mainly due to smaller cohorts experiencing inferior rates of worse outcomes

Regarding hypothalamic integrity, metabolic sequelae were prominent, as evidenced by a postoperative overweight rate of 42.2% (95% CI: 27.6–56.9%). Furthermore, neurocognitive assessment identified postoperative cognitive deficits in 10.6% (95% CI: 6.5–14.6%) of the population. (Fig. 4).

In terms of tumor control during the follow-up period, the pooled rate of recurrence or tumor progression was 14.4% (95% CI: 10.8–18%; I^2^ = 82.91%).

The all-cause mortality rate across the included studies was estimated at 1.9% (95% CI: 1–2.7.7%), with low heterogeneity among studies (I^2^ = 14.69%) (Table 3).

**Table 3:** Outcome data extracted from studies

First author, year	Primary outcome measures				Secondary outcome measures	
	Postop hypopituitarism (*n*)	Postop permanent DI (*n*)	Postop overweight (*n*)	Postop cognitive impairment (*n*)	Recurrence/progression (*n*)	Disease related deaths (*n*)
Bao Y.Y., 2025[16]	183	119	43	6	14	5
Bove I., 2025[17]	50	47	24	8	4	3
Guo Y., 2025[18]	371	204	NA	NA	80	NA
Ke D., 2025[19]	13	8	NA	NA	0	0
Gaillard S., 2024[20]	75	53	2	17	4	0
Chen Y., 2023[21]	16	18	23	1	3	3
Gallotti A. L., 2022[22]	33	30	NA	NA	14	0
Godil S. S., 2022[23]	2	0	3	3	0	0
Zhou Y., 2022[24]	NA	16	47	9	5	1
Zamora R. E., 2022[25]	35	12	NA	NA	15	NA
Zoli M., 2022[26]	65	43	NA	NA	13	0
Chen Z., 2021[27]	NA	25	37	NA	23	0
Duan D., 2021[28]	NA	50	26	NA	10	0
Iglesias P. N., 2021[29]	57	52	49	27/36	NA	NA
Giese H., 2020[30]	NA	33	19	NA	4	12
Simonin A., 2020[31]	8	12	NA	NA	3	1
Cai M., 2019[32]	19	13	1	1	NA	NA
Mou J., 2019[33]	40	23	11	4	1	0
Dho Y. S., 2018[34]	NA	21	21	1	3	2
Zielinski G., 2018[35]	56	44	NA	3	2	NA
Bal E., 2016[36]	8	5	NA	NA	8	0
Fomichev D., 2016[37]	NA	NA	NA	30	22	6
Kshettry V. R., 2016[38]	37	24	22	NA	10	NA
Lee E. J., 2015[39]	69	65	25	25	10	2
Han S., 2014[40]	18	11	NA	0	4	0
Kunihiro N., 2014[41]	13	21	NA	5	2	1
Wang L., 2014[42]	13	1	NA	NA	2	0
Jane J. A. Jr., 2010[43]	10	9	NA	2	2	0
Jung T. Y., 2009[44]	31	29	NA	NA	10	1
Gardner P. A., 2008[45]	13	1	NA	NA	5	NA
Nishizawa S., 2006[46]	20	16	NA	1	2	0
Total (n)	1255	1005	353	116	275	37

### Publication bias assessment

**Fig. 5:**
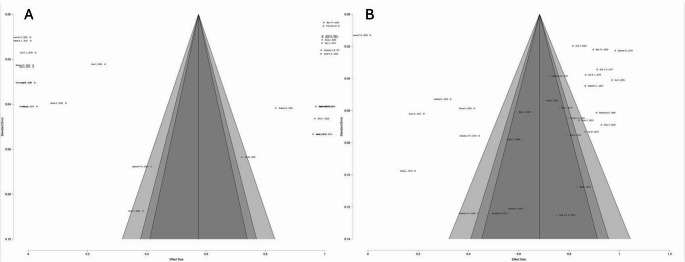
Assessment of publication bias using funnel plots and Egger’s regression test. (**A**) Funnel plot representing the prevalence of the EEEA. The symmetrical distribution of the studies within the funnel indicates an absence of significant publication bias or small-study effects, a finding confirmed by a non-significant Egger’s test (*p* = 0.683). (**B**) Funnel plot GTR rates. The plot exhibits marked asymmetry with a scarcity of studies in the lower-left quadrant, suggesting a bias toward the preferential reporting of positive resection outcomes. This visual assessment is statistically corroborated by a significant Egger’s test (*p* < 0.001)

Assessment of publication bias was conducted using visual inspection of funnel plots and statistically corroborated by Egger’s regression test, utilizing a Restricted Maximum Likelihood (REML) estimator (Fig. 5). The analysis of the EEEA prevalence demonstrated a symmetrical funnel plot distribution, and Egger’s test confirmed the absence of significant bias (*p* = 0.683), suggesting that the reported utilization rates are likely representative of the broader literature. Conversely, the funnel plot for gross total resection rates exhibited marked asymmetry; this was statistically validated by Egger’s test (*p* < 0.001), indicating significant publication bias likely attributable to the preferential reporting of positive surgical outcomes from high-volume tertiary centers.

**Fig. 6:**
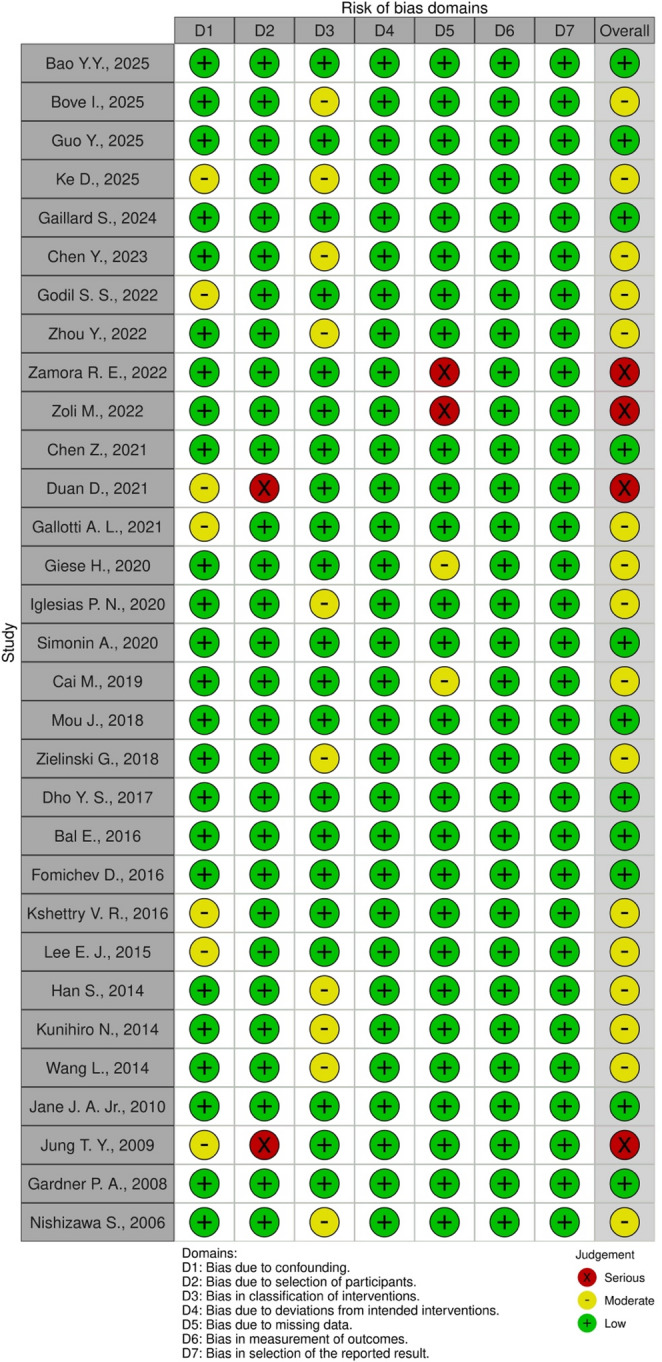
Visualization of qualitative bias assessment with the ROBVIS-I V2 tool

Qualitative assessment of publication bias was conducted with the ROBINS-I V2 tool and visualized with the robvis tool (Fig. 6). In domain 1 (confounding), seven studies [19, 22, 23, 28, 38, 39, 44] were found to be at moderate risk of bias because they did not report on the topography of the tumor, which might reduce the strength of their findings. In domain 2 (classification of intervention), Duan et al.[28] and Jung et al.[44] studies were deemed at serious risk since they did not report the surgical approach employed. We considered 10 studies [17, 19, 21, 24, 29, 35, 40–42, 46] to be at moderate risk of bias in the third domain (selection into the study): the papers in question limited their selection to only one tumor localization or surgical approach, which could introduce some bias in the interpretation of their data. We also considered two studies [25, 26] at serious risk of bias in domain 5 (missing data) since they did not include the extent of resection in their papers. This is fundamental data and its absence impacts the overall analysis. Two more studies [30, 32] were considered to be at moderate risk in domain 5 since they did not report the recurrence rate, but it was not deemed serious due to the aim of this paper. All studies showed a low risk of bias in domain 4 (deviation from intended intervention), domain 6 (measurement of the outcome), and domain 7 (selection of reported results).

## Discussion

### Evolution of surgical approaches

Over the past four decades, the treatment of craniopharyngiomas has evolved from traditional microsurgical craniotomies to endoscopic routes. Early series emphasized the limitations of the microscopic transsphenoidal approach, which was restricted to sellar lesions with limited suprasellar extension. Gardner et al.[45] demonstrated the feasibility of a purely EEEA for suprasellar tumors, achieving NTR or GTR in 91% of patients, with visual improvement in 93% and no new visual deterioration. The study also reported 18% new hypopituitarism and 8% permanent DI, marking the technique as both effective and functionally acceptable despite a high initial CSF leak rate (58%).

Concurrently, transcranial refinements continued to provide reliable access for giant or laterally extended lesions. Han et al.[40] advocated the frontobasal interhemispheric approach, which allowed wide visualization of the third ventricle and suprasellar region with limited retraction. In their 29-patient series, GTR was achieved in 79.3% of cases with visual improvement in 82.7%, new or aggravated hypopituitarism in 13.8%, and permanent DI in 34.4%. This data highlights the approach’s safety for large midline lesions, although carrying the risk of anosmia [47]. Similarly to these trends, Jane et al.[43] reported the EEEA as a valid alternative for adult CPs, achieving 75% GTR by postoperative MRI and ≥ 95% resection in 83% of cases. Visual improvement occurred in 78% of patients, with no postoperative CSF leaks, but permanent DI developed in 44% and new panhypopituitarism in 67%, confirming that endocrine morbidity remains a central challenge.

Several parameters have been analyzed to predict the feasibility of the EEEA for suprasellar lesions, including chiasm position, chiasm-pituitary gland distance, and mammillary bodies-dorsum sellae distance [48]. The latter should be carefully assessed preoperatively, because the mammillary bodies represent the anterior limit of the brainstem, while also playing a crucial role in memory and cognitive functions.

The learning curve for endoscopic skull base surgery was systematically quantified by Kshettry et al.[38], who analyzed 43 EEA cases divided into early and late cohorts. The GTR rate increased from 20% to 65% over time (*p* = 0.005), while major neurological complications dropped from 15% to 4% and CSF leak from 40% to 4% (*p* = 0.007). However, rates of postoperative panhypopituitarism and DI rose significantly due to higher rates of GTR and intentional stalk sacrifice in later years, suggesting a technical trade-off between radicality and endocrine preservation [49]. Similarly, Fomichev et al.[37] compared transcranial and endoscopic approaches, reporting GTR in 74% of EEA cases versus 66% of transcranial resections, with lower visual morbidity and shorter hospital stay in the endoscopic group, confirming the progressive consolidation of EEEA. Nevertheless, Zhou et al.[24] found that a suprachiasmatic translamina terminalis endoscopic approach to intraventricular CP was associated with inferior hypothalamic damage with better functional outcomes, implying that the finer choice of surgical technique plays a significant role in the overall outcomes. Thus, many factors should be taken into consideration when choosing between TCA and EEA (tumor localization, preoperative clinical conditions, skull base anatomy, desired outcomes, and many more) [50].

### Oncological radicality and functional outcomes

The balance between radicality and functional preservation remains a cornerstone of CP surgery. Jung et al.[44] highlighted the role of pituitary stalk preservation, showing that even after stalk preservation, 58% of patients required full hormone replacement, but recurrence-free survival was not negatively affected (recurrence rate 24.4%). Thus, maximal safe resection with stalk preservation remains an optimal target when the tumor allows it.

In our findings, a greater likelihood of GTR compared to non-total resection is evident. We believe that this doesn’t merely represent a publication bias, but reflects the current surgical management of CPs, since GTR is the main predictor of progression-free survival [51], and the EEEA implementation has increased rates of total resection [52]. This, in turn, led to the low recurrence rate of 14.4% we found in this review. Nevertheless, the increased rates of total resection have been associated with an increased probability of negative postoperative neuroendocrinological and cognitive outcomes due to damage to the hypothalamic-pituitary structures [53]. Indeed, our analysis found a significant postoperative increase in neuroendocrinological and cognitive dysfunctions, with 79.6% of hypopituitarism, 54.7% of DI, 42.2% of obesity, and 10.6% of cognitive decline (the latter probably underestimated by underinvestigating and underreporting). Endocrinological morbidity remains the dominant postoperative concern regardless of the approach, especially considering the different tumor locations. Indeed, suprasellar localization predicts hypothalamic involvement [54], which in turn is related to increased functional deficit rates. Nishizawa et al.[46] demonstrated that resecting the stalk more distally is associated with better preservation of posterior pituitary function, but worse outcomes of the anterior gland. This knowledge may be useful in patients with an already compromised anterior function but a preserved posterior gland.

Visual outcomes remain a strength of endoscopic techniques. Gardner [45], Han [40], Kunihiro [41], and Kshettry [38] all reported > 80% rates of visual improvement and < 5% of new visual deterioration.

It has to be noted that a significant number of studies did not report preoperative cognitive impairment rates, and that there is a general lack of standardization in neurocognitive outcomes for CP patients. Interestingly, Giese et al.[30] prospectively evaluated 36 patients from a neuropsychological point of view and discovered how TCAs negatively impact cognitive functions more than the EEEA. Amongst TCAs, the subfrontal approach is the most detrimental. The side of the approach is also associated with different cognitive outcomes: left-sided pterional craniotomies determined worse long-term intellectual performances compared to right-sided approaches. Moreover, intraventricular and larger CPs show worse cognitive impact. They attribute these findings to the retraction applied on the brain during TCAs and to the hypothalamic invasion of larger and intraventricular CPs. Although the study is well conducted, with patients prospectively enrolled and examined, the authors do not report on radiotherapy or the extent of resection pursued on those patients. These factors may play a role in intellectual performance and influence test results.

Postoperative weight gain is another critical outcome to evaluate in CP surgery. Its development is likely multifactorial, depending both on hypopituitarism and mediobasal hypothalamic damage resulting in loss of energy homeostasis [55]. Duan et al. showed how a worse preoperative BMI was paradoxically linked to a less severe increase in the postoperative period, likely reflecting an already damaged hypothalamus or the curative effect of CP removal. Indeed, weight gain is linked with worse pituitary function and increased rates of DI[56], confirming the central interconnected role of the hypothalamus in CP surgery. Regrettably, from our analysis, there is a clear lack of reporting of intraoperative hypothalamic infiltration by CPs, even though it is becoming increasingly clear how their relationship significantly influences resection rates and functional outcomes [57]. This underreportage reduces the possibility for more thorough discussion of functional outcomes, together with the modest heterogeneity found in the data gathered in this review. As highlighted in Fig. 4, the main source of heterogeneity comes from smaller series, reporting inferior rates of worse functional outcomes. This partly depends from smaller centers reporting on their experience on CP surgery, likely mirroring less complex cases as compared to higher volume centers, ultimately reducing the overall generalizability of their findings.

### Oncologic control, technical innovations, and future directions

Long-term control remains primarily dependent on the extent of resection.

Bal et al.[36] also emphasized the oncologic advantage of surgical resection combined with adjuvant radiotherapy for residual disease, noting that conservative subtotal resections were associated with higher recurrence despite lower immediate morbidity. Advances in vascularized flap reconstruction, intraoperative endoscopy, and neuronavigation have dramatically reduced CSF leak rates compared to early endoscopic series. Kshettry et al.[38] demonstrated a drop from 40% to 4% following adoption of the Hadad–Bassagasteguy flap.

Meanwhile, the integration of molecular therapy (e.g., BRAF/MEK inhibitors for papillary variants [58]) has begun to influence preoperative planning and may soon complement surgery for function-preserving strategies. Moreover, advanced radiotherapeutic modalities are being increasingly adopted for CPs. Recent data shows that STR followed by radiotherapy achieves similar progression-free rates to GTR: the literature shows that the pooled hazard ratio for the two different management strategies do not significantly differ [59, 60]. Conservative multimodal strategies also offer inferior rates of functional impairments: GTR is associated with 54.8% and 56.3% rates of panhypopituitarism and DI respectively, against the 26.7% and 13.3% associated with SRT+RT [61]. This is probably due to the possibility of better defining tumor treatment volumes with new technologies. Proton therapy is also emerging as an increasingly attractive option thanks to the possibility for radiation modulation inside tumor volumes [62], granting increased safety for surrounding healthy tissue and better hippocampus-sparing strategies [63, 64]. Rutenberg et al. showed a 100% local control rate at 3 years post-proton therapy [65]. Even though the population was small (14 patients), we believe this evidence is noteworthy. In a population of 77 patients, Jimenez et al. reported a 92% rate of disease control after a mean 4.8 years, with no worsening of cognitive abilites [66].

While authors show a general sensitivity for hypothalamic-sparing strategies, no standardized approach to CPs is found in the literature.

In our experience, especially for earlier stages of the disease in younger patients, GTR should not be pursued at the cost of hypothalamic demolition The negative neuroendocrinological outcomes on patients’ health may sometimes determine a worse impact than the disease itself, especially considering the biologically benign nature of CPs. Given the new adjuvant treatments emerging, except in cases of biologically aggressive CPs, we usually prefer a conservative, safe maximal resection with strict postoperative radiological follow-up and adjuvant radiotherapy in cases of recurrence or progression, as advised by the consensus paper published by the European Association of Neurosurgical Societies (EANS) [67].

## Limitations

Despite significant advances, current evidence remains dominated by retrospective, single-institution experiences with variable criteria for hypothalamic involvement and incomplete functional follow-up. Heterogeneity in reporting of GTR, hypothalamic dysfunction, and endocrine outcomes reduces meta-analytic precision, as well as differences in data reporting across studies. Additionally, publication bias persists, with overrepresentation of high-volume centers reporting superior oncological outcomes while lower-volume centers report inferior rates of postoperative functional impairments. This limits the generalisability of their claims and should be carefully interpreted.

Furthermore, the comparison between EEEA and TCA is inherently confounded by tumour topography and hypothalamic involvement. Since the EEEA is predominantly utilized for midline and suprasellar lesions, whereas transcranial routes are often required for giant or laterally extended tumors, these anatomical factors strongly influence both surgical approach selection and functional outcomes. This inherent bias limits the accuracy of direct, approach-based comparisons and must be carefully considered when interpreting the overall results. Lastly, our selection criteria (patient age > 15 years) might introduce some bias, not representing a strictly adult population, although homogeneous from a management point of view.

##  Conclusions

 Recent literature demonstrates that the EEEA is being increasingly favored for midline and suprasellar CPs, achieving robust rates of GTR with acceptable morbidity when performed in experienced hands. A general underreporting of hypothalamus infiltration at surgery is evident, underestimating its significance in CP surgery and making the evaluation of functional outcome incomplete. Prospective, multicentric studies are extremely needed to better assess surgical outcomes, especially considering new treatment options that are showing comparable outcomes with inferior morbidity.

## Data Availability

No datasets were generated or analysed during the current study.
